# Gut Dysbiosis Thwarts the Efficacy of Vaccine Against *Mycobacterium tuberculosis*

**DOI:** 10.3389/fimmu.2020.00726

**Published:** 2020-05-19

**Authors:** Sajid Nadeem, Sudeep K. Maurya, Deepjyoti K. Das, Nargis Khan, Javed N. Agrewala

**Affiliations:** ^1^CSIR-Institute of Microbial Technology, Chandigarh, India; ^2^Indian Institute of Technology, Ropar, India

**Keywords:** vaccine, BCG, L91, antibiotics, dysbiosis, memory T cells

## Abstract

The generation of enduring protective immunity by vaccines is of utmost importance. Intriguingly, there is considerable variation in the efficacy of vaccines amongst individuals. Various studies have shown that normal flora of gastrointestinal tract plays a vital role in maintaining host homeostasis and immunity. Since gut microbiome is also extremely variable between individuals, we speculate that it might impact individual’s response to vaccines. Consequently, we administered broad spectrum antibiotics cocktail to induce gut dysbiosis and monitored its impact on the generation of long-lasting memory T cells and thereby BCG vaccine efficacy. Interestingly, gut dysbiosis significantly decreased the activation of CD4^+^ T cells and CD8^+^ T cells. Further, there was decline in the frequency of memory CD4^+^ T cells and CD8^+^ T cells in lungs and secondary lymphoid organs of the vaccinated animals. Moreover, it dampened the IFN-γ and TNF-α secretion and proliferation of *Mtb*-specific T cells. Most importantly, dysbiosis hampered *Mtb* clearance in vaccinated animals, as evidenced by increase in the colony forming units (CFUs) in lungs and spleen. Our findings indicate that gut dysbiosis can be one of the major factors responsible for variable efficacy of TB vaccines across the world.

## Introduction

Tuberculosis (TB) has been a modern scourge for mankind immemorial. *Mycobacterium tuberculosis (Mtb)*, the etiologic agent of TB was first identified in 1882; however, this global burden has not diminished to date. According to the global tuberculosis report 2017, there were approximately 1.3 million deaths due to TB worldwide. Although, 90–95% of the population infected with *Mtb* remains protected throughout their life span but 5–10% people still develop an active TB disease. At present, the only available vaccine for TB is Bacillus Calmette Guerin (BCG) which is effective in neonates and young children but not so efficacious for adult pulmonary disease. Currently, the only TB treatment available for patients is the standard drug regimen of isoniazid and rifampicin drugs. Drug therapy has become inefficient due to emergence of multidrug and extremely drug resistant strains of the *Mtb*. Since 1928, almost 4 billion people have been vaccinated around the globe with BCG (1). Due to its inconsistent performance worldwide, BCG could not impart adequate protection; and therefore failed to reduce the TB burden. Many reasons have been proposed to explain the failure of BCG. First is the interference by non-tuberculosis mycobacteria (NTM) in the BCG-mediated immune response, which can induce immune regulation (2). Second is the obstruction in the antigen processing and presentation by mycobacteria, and third is skewing of BCG induced response by helminths (3, 4).

In recent years, there has been an array of studies highlighting the importance of gut microbiota in influencing the health and disease (5). Further, gut microbiota can modulate the immune system not only at enteric sites but also at systemic sites (6, 7). Although, the advent of antibiotics in last century has led to sharp decline in mortality from infectious diseases but the inappropriate use of antibiotics has resulted in drug resistance, especially in the case of TB (8). Many reports have stated that usage of antibiotics alter the gut microbiota significantly, especially in children and neonates when the relationship between gut microbiota and host immune system is established (9–11). Recently, we have shown that disturbance in gut microbiota composition increases the host susceptibility to TB (12). Consequently, the interplay between altered gut microbiota and vaccine efficacy, which is also given in childhood cannot be ignored.

Considering above-mentioned facts, this study was designed to monitor the influence of perturbation of gut microbiota in the efficacy of BCG vaccine. Interesting observation noted was that gut dysbiosis impaired the efficacy of BCG in vaccinated animals, as evidenced by impaired generation of effector and memory T cells and increase in colony forming units (CFUs) in lungs of *Mtb* infected mice. This study signifies that gut dysbiosis can substantially affect the endurance and protective efficacy of vaccines.

## Materials and Methods

### Animals and Ethics Statement

BALB/c mice, 6–8 weeks used in the experiments were procured from Animal House Facility, Institute of Microbial Technology (IMTECH), Chandigarh. Use of animals was approved by the Institutional Animal Ethics Committee of IMTECH and experiments were performed in accordance with the National Regulatory Guideline issued by Committee for the Purpose of Supervision of Experiments on Animals (No. 55/1999/CPCSEA), Ministry of Environment and Forest, Government of India.

### Mycobacterial Strain and Antigens

*Mycobacterium tuberculosis* virulent strain *H37Rv* was generously provided by Dr. VM Katoch, National JALMA Institute for Leprosy and Other Mycobacterial Diseases, Agra, India. *Mtb* was cultured in Middlebrook 7H9 broth containing glycerol (0.2%) and Tween-80 (0.05%) which was supplemented with 10% oleic acid, albumin, dextrose and catalase (OADC). The viability of bacteria was determined by plating on Middlebrook 7H11 medium, containing 0.5% glycerol and supplemented with 10% OADC. Plates were kept in incubator at 37^o^C and numbers of CFUs were counted after 21 days of plating.

### Chemicals and Reagents

All chemicals and reagents were purchased from Sigma Aldrich (St. Louis, MO, United States) unless otherwise mentioned. For culturing of cells, tissue culture grade plastic ware was purchased from BD Biosciences (Bedford, MA, United States). RPMI-1640 media and fetal bovine serum (FBS) were purchased from GIBCO (Grand Island, NY, United States) for cell culture. L-pyruvate, L-glutamine, streptomycin and penicillin were from Serva (Heidelberg, Germany). Anti–mouse fluorochrome labeled antibodies (Abs): CD4-PB, CD62L-APC, CD44-PerCP-Cy5.5, CD127-PE, and Abs for ELISA were procured from BD Pharmingen (San Diego, CA, United States) or otherwise mentioned.

### Vaccine Construct Used in Study

Lipidated synthetic peptide vaccine construct used in the study was prepared using solid phase synthesis method which has been detailed elsewhere (13). The promiscuous peptide sequence “SEFAYGSFVRTVSLPVGADE” used in the construct was derived from Acr1 protein of *Mtb.* It was linked to Pam2Cys which specifically binds to TLR2 receptor on DCs. This binding activates the DCs, which in turn activate T cells.

### Study Design

Mice were vaccinated with BCG (1 × 10^6^ CFU) or L91 (20 nmol) vaccine candidates at the base of tail through subcutaneous (s.c.) route. A booster dose of 10 nmol was given after 15 days of primary immunization. After 60 days of secondary immunization, mice were fed with antibiotics (Abx) cocktail *ad libitum* in drinking water for 21 days and thereafter fecal CFUs were performed. Abx cocktail used in the study were amphotericin B (50 mg/L), trimethoprim (20 mg/L), polymyxin B (60 mg/L), vancomycin (100 mg/L) and carbenicillin (50 mg/L) (12). Drinking water containing Abx was replaced after every 5 days. Control mice were fed with water without Abx. Subsequently, after 21 days, mice were aerosol challenged with 100 CFU of *Mtb* deposition in lungs using the aerosol machine Inhalation Exposure System (Glas-Col, LLC, Terre Haute, IN, United States). The animals were administered Abx for another 21 days (total Abx: 42 days) post-*Mtb* challenge. The administered Abx cocktail used in the study did not affect the viability of *Mtb* (checked by plaque forming unit: Data not shown), so we continued the Abx treatment post-*Mtb* challenge to make sure that the disrupted gut microbiota remains the same and not restored. Later, mice were sacrificed and tissues were aseptically harvested for various microbiological and immunological studies.

### Isolation of Lymphocytes From Spleen, Lymph Nodes, and Lungs

Experimental mice were sacrificed and their spleen, lymph nodes and lungs were collected. Subsequently, spleen and lymph nodes were pooled and single cell suspension was prepared by pressing through frosted slides. The lungs were perfused using cold 1× PBS containing heparin (100 U/ml). Thereafter, lung tissue was minced to small pieces and digested with collagenase (2 mg/ml) and DNase (0.03 mg/ml) for 30 min at 37°C. Subsequently, cells were passed through a sieve (70 μm) to prepare single cell suspension of lung tissue. Removal of red blood cells (RBCs) was done using ACK lysis buffer followed by three washes with 1× PBS. Subsequently, the cells were suspended in RPMI-1640 media supplemented with 10% FBS. Cell viability was checked by trypan blue dye exclusion method.

### *In vitro* Stimulation With Antigen

Single cell suspension obtained from lungs, spleen and lymph nodes of vaccinated animals (2 × 10^5^/well) were added to 96 well U-bottom culture plates and cultured with *Mtb*- specific purified protein derivative (PPD; 20 μg/ml) or L91 (1 nmol) for 48 h at 37°C/5% CO_2_ (14, 15). Later, these cells were harvested and used for immunological phenotype assessment using flow cytometer and cytokine estimation by ELISA in culture supernatants.

### Flow Cytometric Analysis

For cell phenotypic analysis, briefly, 5 × 10^5^ lymphocytes were collected in tubes followed by washing with 1× PBS supplemented with 2% FBS once. Cells were then treated with Fc receptor blocking Ab followed by staining with fluorochrome conjugated anti-CD4, CD8, CD44, CD62L, and CD127 Abs as per the recommended protocol. The cells were acquired in BD FACS ARIA and analysis was performed using DIVA software. The gating strategy used for the flow cytometry analysis is provided (Supplementary Figure 3).

### Proliferation Assay

Equal number of single cell suspensions of lungs were stained with either carboxyfluorescein succinimidyl ester (CFSE) dye (1 μM) or efluor 670 dye (1 μM) by incubating in phosphate-buffered saline (PBS) at 37^o^C for 10 min. Free dye was quenched using 2 ml of FCS and washed 3 times with RPMI-FCS-10%. Subsequently, equal number of stained cells from both groups were re-stimulated with *Mtb* PPD (20 μg/ml) or L91 (1 nmol) for 48 h to expand *Mtb-* specific T cells. Later, the cells were gated on CD4^+^ T cells and CD8^+^ T cells and proliferation was monitored by flow cytometry. Absolute number of proliferating cells were back calculated and plotted as bar graph per 200,000 cells analyzed.

### Quantification of Cytokine Secretion by ELISA

The secretion of cytokines in culture supernatants (SNs) was estimated by sandwich ELISA as per standard protocol. ELISA plates were coated with anti-IFN-γ and TNF-α Abs overnight at 4^o^C. Next day, the plates were washed with PBST buffer (1× PBS + 0.05% tween-20) to remove excess unbound antibody and unbound sites were then blocked with 1% BSA. Subsequently, culture supernatants were added along with the respective standards of cytokines. Following overnight incubation, samples were treated with biotinylated detection antibody for 2 h. Later, plates were washed properly with PBST to remove unbound antibody and incubation with streptavidin-HRP was done for 1 h at room temperature (RT). After that, OPD chromogen substrate was added for development of color. The reaction was stopped using 7% H_2_SO_4_ and absorbance was measured at 492 nm. The quantification of secreted cytokines in cultures was done using standard plot generated from the recombinant cytokine of known concentration and was expressed as ng/ml.

### *In vivo Mtb* Survival Assay

Animals were aerosol challenged with *Mtb*-*H37Rv* as described previously. After 21 days of aerosol challenge, the *Mtb* burden in the lungs and spleen of animals was assessed by serially diluting the lung and spleen homogenates and plating them on Middlebrook 7H11 agar plates supplemented with 10% OADC enrichment and an Abx cocktail of polymyxin B sulfate, vancomycin, amphotericin, carbenicillin, trimethoprim and cycloheximide. Plates were monitored weekly and CFU count was recorded after 18–20 days of incubation at 37^o^C.

### Fecal Sample Collection and DNA Extraction

Isolation of fresh fecal pellets was done before mice were euthanized. These pellets were collected in sterile conditions and immediately preserved in −80°C. Immediately, after sacrificing the mice, contents of the cecum and parts of distal small intestine (ileum) were also collected and preserved for DNA isolation. The collected fecal pellets were processed and total DNA was extracted using ZR Fecal DNA MicroPrep kit (South San Francisco, CA, United States) as per manufacturer’s instruction. Finally, DNA was eluted in 20 μl elution buffer and DNA was quantified by NanoDrop spectrophotometer. Purity of the DNA in all samples was assessed by monitoring the A_260_/A_280_ ratio which was in the range of 1.7–1.8.

### Cultivable Microbes

For cultivation of microbes, fecal samples (250–300 mg) were collected aseptically in 1 ml of 1× PBS. After homogenizing these samples, they were centrifuged at 3000 rpm for 2 min to settle down the debris and SNs was collected. Subsequently, serial dilutions of the collected SNs were made and 100 μl were plated on different agar media to culture both aerobic and anaerobic microbes. The cultivation of anaerobic microbes was performed by incubating the plates in presence of anaerobic gas pack (Himedia, Mumbai, India) in vacuum tight containers for overnight at 37^o^C.

### Assessment of Bacterial Diversity

The assessment of bacterial diversity was done as described elsewhere (12). Briefly, supernatant from fecal samples were plated on different media to culture both aerobic and anaerobic microbes for overnight as briefed above. Four different parameters were selected for identification of colony morphotypes- colony size, texture, color, and form. A phenotypic variant was considered when it differed in at least one of the above- mentioned morphological parameters. Assessment of total diversity was done by counting total number of different colonies grown on different media plates. A reference number was considered based on the different number of colonies obtained in feces of healthy animals. The decrement in fecal diversity was calculated using the formula: [(reference number - total different colonies counted from infected or antibiotics treated animals)/reference number] × 100.

### Reconstitution of Gut Microbiota

In order to restore the gut flora, Abx treated animals underwent fecal transplant. To accomplish this, fecal samples (200–300 mg) were collected from healthy mice under aseptic condition in 1ml of 1× PBS. The pellets were homogenized and then centrifuged at 2000 rpm for 3 min. The resulting supernatant slurry was collected and 100 μl was given to mice through oral gavage within 10–15 min of excretion. Four such doses were orally administered to mice with a gap of 4-day interval prior to sacrificing animals.

### Quantification of Gut Microbes by RT-qPCR

Isolation of DNA from fecal samples was performed using ZR Fecal DNA Microprep kit, as per the manufacturer’s instructions (Epigenetics, Irvin). Quantification of DNA was done with the help of NanoDrop spectrophotometer. Ratio of A_260_/A_280_ for all samples was in the range of 1.7–1.8. RT-qPCR was performed in a final volume of 10 μl, constituting of 1× SYBR green, 50–100 ng of DNA, 0.2 μM forward primer and 0.2 μM reverse primer. PCR reactions were run and analyzed on real-time PCR system (StepOnePlus; Applied Biosystems, Waltham, MA) according to manufacturer’s instruction. Analysis was done by comparative Ct method, whereas Ct values were normalized against universal control. Expression unit were expressed as 2^–(Δ^ ^*CT*×10000)^. Following primer sequences were assessed:

All Bacteroides Fwd 5′-GAGAGGAAGGTCCCCCAC -3′
All Bacteroides Rev 5′-CGCTACTTGGCTGGTTCAG-3′
All Bifidobacteria Fwd 5′-GTCCGTGACCTCCTCGAC-3′
All Bifidobacteria Rev 5′-GTGGAAGGTCTCGATGGAG-3′
All Lactobacillus Fwd 5′-AGCAGTAGGGAATCTTCCA-3′
All Lactobacillus Rev 5′-CACCGCTACACATGGAG-3′
All Universal primer Fwd 5′TGGAGCATGTGGTTTAAT TCGA3′
All Universal primer Rev 5′TGCGGGACTTAACCCAACA3′

### Statistical Analysis

Statistical analysis was done using one-way analysis of variance (ANOVA) with post Tukey-Kramer multiple comparisons test unless otherwise mentioned using Graph Pad Prism 6 software. Statistical significance was expressed as ^∗^*p* < 0.05, ^∗∗^*p* < 0.01, ^∗∗∗^*p* < 0.001. Only *p* < 0.05 were considered statistically significant.

## Results

### Treatment With Abx Significantly Alters Gut Microbial Composition and Influences the Efficacy of TB Vaccines

**FIGURE 1:**
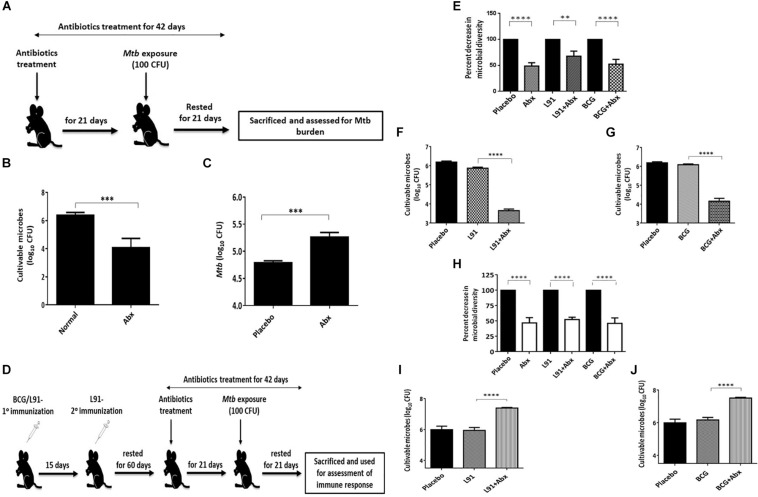
Abx distorted the gut microbial composition. **(A)** Schematic representation of a mouse model to evaluate the influence of Abx on gut microbiota. Mice were administered Abx for 21 days. **(B)** Subsequently, 21 days post Abx treatment, frequency of cultivable microbes was monitored in the fecal samples by CFU assay. The control group was administered PBS only (Normal). Later, the animals were *Mtb* challenged with 100 CFUs deposition in the lungs. **(C)** The *Mtb* burden in the lungs was monitored after 21 days. The control group was administered PBS (Placebo). **(D)** Mice were injected with BCG (1 × 10^6^ CFU) or L91 (20 nmol) vaccine candidates and then rested for 60 days. Subsequently, mice were administered Abx for 42 days. After the initial Abx treatment for 21 days, the animals were challenged with *Mtb*. Cultivable microbes and microbial diversity were monitored in the fecal samples on day 5 **(E–G)**; day 42 **(H–J)** of Abx treatment by CFU assay on different agar media. Data shown as mean ± SEM are representative of 2 independent experiments (*n* = 3–4 animals/group). **p < 0.01, ***p < 0.01, and ****p < 0.0001.

Alteration of gut microbial composition has been shown to have a significant effect on the immunological response generated in body. It not only modulates the immune response at local sites but also affects host immune response at systemic sites. Therefore, we were curious to know whether gut dysbiosis can modulate the efficacy of vaccine. Abx treatment has already been shown to alter the intestinal microbial composition (16). To test our hypothesis, firstly we used a combination of broad spectrum Abx for depletion of intestinal microbial flora; and alteration in the microbiota was assessed by CFUs plating (Figure 1A). After 21 days of Abx treatment, it was observed that the mice showed a markedly reduced number and diversity of microbial flora, as exemplified by a significantly [p < 0.001] reduced number of microbial taxa (Figure 1B). After monitoring the Abx mediated disruption of microbiota, we were interested in evaluating the *Mtb* survival in the lungs of these animals. After 21 days of Abx treatment, animals were aerosol challenged with *MtbH37Rv*. Subsequently, after 21 days, bacterial burden in the lungs of these animals was assessed and it was observed that animals with disrupted gut microbiota showed a significantly higher *Mtb* burden in the lungs (Figure 1C). The result clearly indicated that gut dysbiosis hampers the systemic immunity and thus have a significant effect on the host’s response to pathogen. Next, we wanted to determine the effect of gut microbiota disruption on the efficacy of TB vaccines *viz*, BCG and L91. BCG is the most widely accepted and WHO approved vaccine for tuberculosis. L91, on the other hand is a peptide- based vaccine, which has shown promising result in reducing *Mtb* burden in both mice and Guinea pig models of TB than BCG (17). To evaluate the effect of Abx treatment on the vaccinated mice, mice were initially vaccinated with BCG and L91. After 60 days of vaccination, animals were *ad libitum* fed with Abx and were continued for next 42 days (Figure 1D). The initial disruption of gut flora was assessed after 5 days by CFU plating. Expectedly, we observed a significant decrease (*p* < 0.0001) in the microbial number, as well as diversity in Abx treated animals, as compared to non-Abx treated mice that were vaccinated with L91 and BCG (Figures 1E–G). After 21 days of Abx treatment, animals were aerosol challenged with *Mtb* and microbial number and diversity was again checked in these animals, 42 days post Abx treatment. A significant increase (p < 0.0001) in microbial number was observed but the diversity remained sparse (Figures 1H–J). This observation suggested that microbial flora, which escaped the Abx mediated elimination repopulated at later time point and occupied the whole niche and subsequently restored the microbial number but overall microbial diversity was decreased (Figures 1H–J).

### Gut Dysbiosis Using Abx Significantly Hampers the Proliferation and Activation of Antigen Specific CD4^+^ T Cells and CD8^+^ T Cells in Vaccinated Animals

**FIGURE 2:**
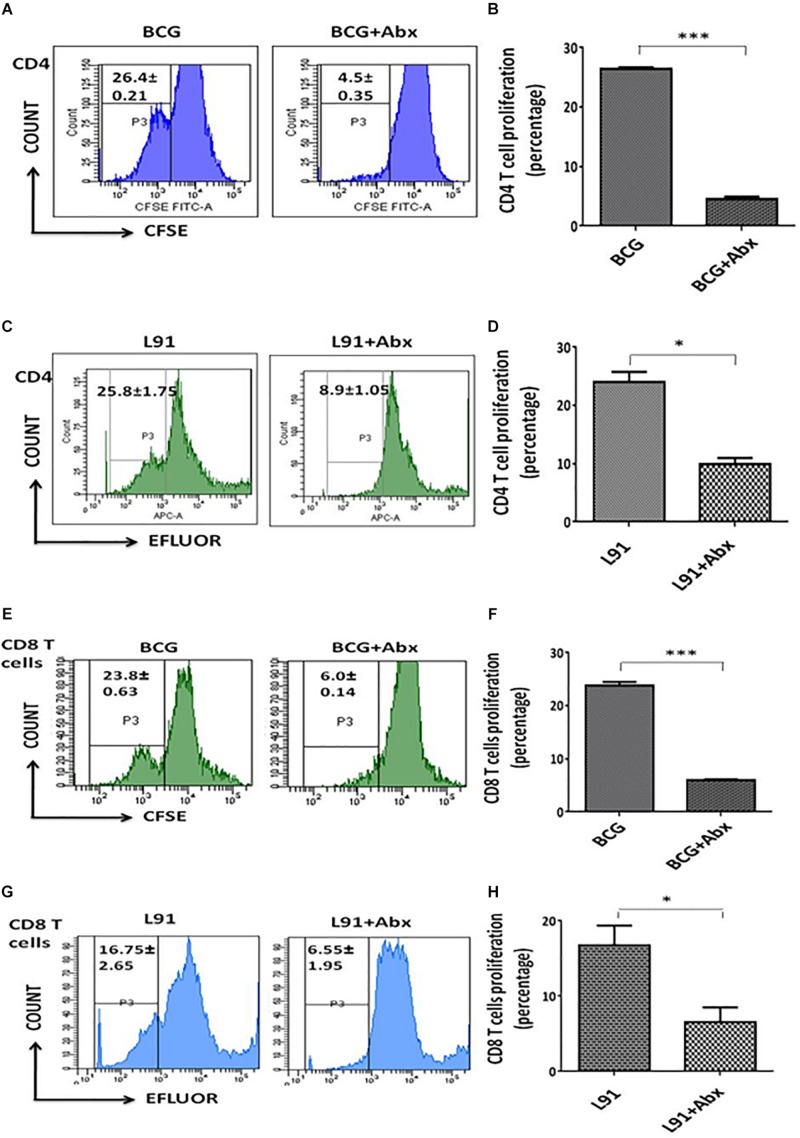
Dysbiosis of gut microbiota in vaccinated animals hampered the proliferation of T cells. After 21 days of *Mtb* exposure, the lungs were isolated from the BCG and L91 vaccinated and Abx treated mice. Single cell suspensions of lung cells were prepared and equal number of cells in both groups were stained with CFSE/efluor dye. Subsequently, the cells were *in vitro* stimulated with PPD for 48 h. Later, the cells obtained from **(A,B,E,F)** BCG and **(C,D,G,H)** L91 vaccinated mice were subsequently gated on **(A–D)** CD4^+^ T and **(E–H)** CD8^+^ T cells and monitored for the proliferation. The number in histogram indicates the percent of (CFSE/efluor)^*lo*^ population. Data shown as mean ± SEM are the representative of 2 independent experiments (*n* = 3–4 animals/group). *p < 0.05, ***p < 0.001.

T cells are the cardinal cells of cellular mediated immunity and a major player in imparting protection against *Mtb*. In order to understand the effect of Abx on *Mtb* survival and immune response, firstly we monitored the antigen specific proliferative ability of T cells in Abx treated and vaccinated groups. Lymphocytes (CD4^+^ T cells and CD8^+^ T cells) isolated from BCG and L91 immunized animals were stained with CFSE dye for monitoring clonal expansion of T cells on restimulating with *Mtb-* specific PPD. Interestingly, CD4^+^ T cells (*p* < 0.001) and CD8^+^ T cell (*p* < 0.001) isolated from ^Abx^BCG vaccinated animals (Abx treated and BCG vaccinated animals) showed substantially diminished proliferation, as compared to non- Abx and vaccinated animals (Figures 2A,B,E,F and Supplementary Figures 4A,B). Similarly, CD4^+^ T cells (*p* < 0.05) and CD8^+^ T cell (p < 0.05) of ^Abx^L91 vaccinated animals (Abx treated and L91 vaccinated animals) exhibited subdued proliferation as compared to their normal counterparts (Figures 2C,D,G,H and Supplementary Figures 4C,D). This suggested that Abx mediated gut dysbiosis impairs the proliferative ability of T cells.

**FIGURE 3:**
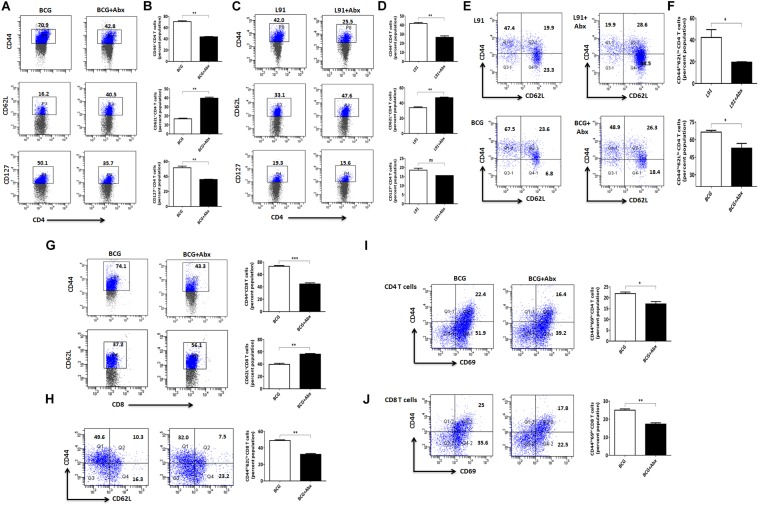
Abx mediated gut dysbiosis impaired the activation and generation of memory T cells in lungs. The cells isolated from the lungs of ^Abx^BCG or ^Abx^L91 vaccinated mice were cultured and *in- vitro* stimulated with PPD for 48 h. After 48 h, the cells were harvested, stained and gated on **(A–F,I)** CD4^+^ T cell; **(G,H,J)** CD8^+^ T cells and assessed for the **(A–D)** activation markers CD44, CD62L, CD127, and CD69; **(E,F)** effector memory CD44^hi^CD62L^*lo*^ (T_EM_) and (I–J) Resident memory CD44^hi^CD69^hi^ (T_RM_). Values in the inset and bar graphs signify percent population. Data shown as mean ± SEM are representative of two independent experiments (*n* = 3–4 animals per group). *p < 0.05, **p < 0.01, ***p < 0.001.

Next, we assessed the activation status of the CD4^+^ T cells and CD8^+^ T cells in lymphocytes isolated from lungs of vaccinated animals. The ^Abx^BCG animals showed a significantly diminished activation profile of T cells as compared to non-Abx vaccinated groups, as evidenced by reduced expression of activation markers CD44 (*p* < 0.01) and CD127 (*p* < 0.01) and enhanced expression of CD62L (*p* < 0.01), which is an indicator of naïve T cell population (Figures 3A,B). Similar trend was observed in case of ^Abx^L91 animals which displayed a substantially decreased population of CD44 (*p* < 0.01) and CD127 (*p* < 0.01) and enhanced CD62L (*p* < 0.01) population (Figures 3C,D). In addition, lung cells isolated from L91 vaccinated animals showed similar trend when *in vitro* stimulated with L91 peptide for recall response (Supplementary Figure 5). Moreover, CD8^+^ T cells isolated from Abx-treated and BCG vaccinated animals exhibited reduced expression of CD44^+^ (*p* < 0.001) and enhanced expression of CD62L (*p* < 0.01) as compared to non-Abx BCG vaccinated group (Figure 3G). The results clearly indicated that gut dysbiosis affected the systemic immune response by hampering the activation of CD4^+^ T cells and CD8^+^ T cells.

### Abx Mediated Dysbiosis Hampers the Memory T Cells and Lung Resident T Cell (T_RM_) Population

Success of any vaccine relies on the generation of a long-lasting memory T cell population, which is extremely crucial to respond to future encounter with pathogen. As a result, we monitored the effect of gut dysbiosis on generation of long-lasting memory T cell in lungs. Lymphocytes isolated from lungs were stained for memory T cell markers (CD44 and CD62L) on CD4^+^ and CD8^+^ gated T cells. Interestingly, ^Abx^BCG and ^Abx^L91 groups showed a considerably reduced effector memory T cell population on CD4^+^ T cells [BCG (*p* < 0.05); L91 (*p* < 0.05)] and CD8^+^ T cells [BCG (*p* < 0.01)], as expressed by CD44^hi^CD62L^*lo*^ population in lungs of dysbiosis induced animals, when compared to non-Abx treated groups (Figures 3E,F,H).

Resident memory T cells (T_RM_) are an important subset of memory T cells that plays an important role in providing frontline defense against re-encounter of pathogens (18). Unlike T effector memory cells (T_EM_) and T central memory cells (T_CM_), T_RM_ do not circulate between blood and tissues but remain localized in the tissues. The role of T_RM_ has been widely studied in viral and bacterial infection where they recruit and activate immune cells (19–22). During *Mtb* infection, there is a significant generation of lung-tropic CD4^+^ T_RM_, which mediates clearance of pathogens and imparts protection (23–25). Considering the immense significance of T_RM_ in host immunity, we checked the impact of gut dysbiosis on the T_RM_ population. There was a drastic decrease in the population of CD4^+^ T_RM_ [BCG (*p* < 0.05)] and CD8^+^ T_RM_ [BCG (*p* < 0.01)] in lungs of dysbiosis induced animals (Figures 3I,J). The reduction in the lung T_RM_ is an indicator of subdued immune response generated in dysbiosis induced animals.

### Dysbiosis of Gut Flora Suppresses Activation of T Cells at Secondary Lymphoid Organs

**FIGURE 4:**
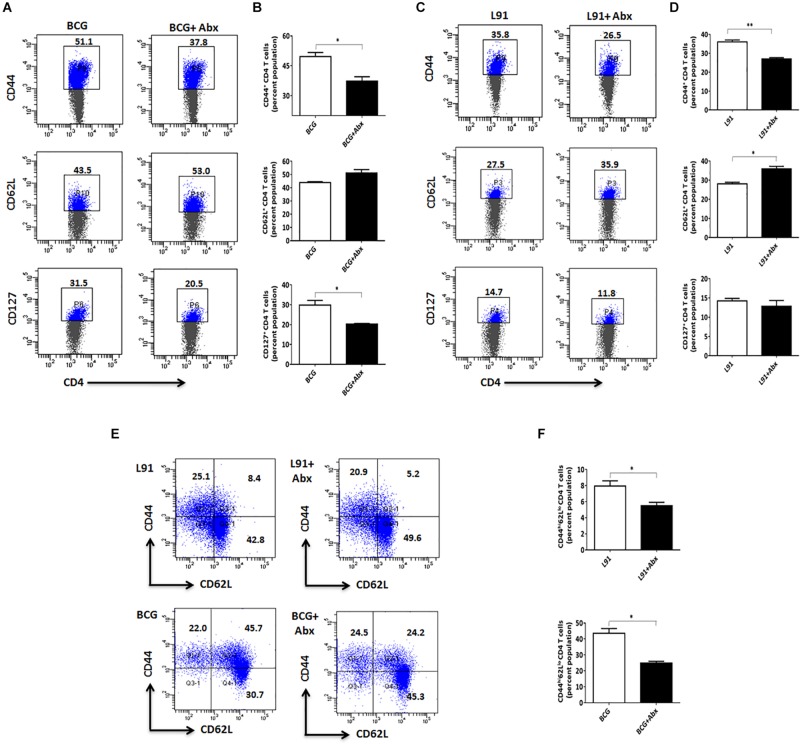
Gut dysbiosis hampers the activation and generation of memory T cells in secondary lymphoid organs. The cells isolated from the spleen and lymph nodes of Abx treated and BCG or L91 vaccinated mice were cultured, as indicated in the legends of Figure 2. The cells obtained were gated on CD4^+^ T cells and assessed for the **(A–D)** activation markers CD44, CD62L, and CD127; **(E,F)** central memory population CD44^hi^CD62L^hi^ (T_CM_). Values in the inset and bar graphs denote percent population. Data shown as mean ± SEM are representative of two independent experiments (*n* = 3–4 animals per group). *p < 0.05, **p < 0.01.

After monitoring the subdued activation of T cells in lungs, we were curious to monitor the status of T cells in secondary lymphoid organs. Activation of T cells at these sites is of utmost importance for imparting efficient protection against pathogens. The animals were sacrificed and splenocytes were cultured *in vitro* with *Mtb*-specific PPD for 48 h to assess *Mtb-*specific T cell response. Interestingly, there was a marked reduction in the percentage of activated T cells obtained from ^Abx^BCG immunized animals compared to BCG, as evidenced by diminished CD44 (*p* < 0.05) and CD127 (*p* < 0.05) expressing population and enhanced CD62L population (Figures 4A,B). Similarly, ^Abx^L91 immunized animals exhibited subdued CD44 (*p* < 0.01) and CD127 displaying cells and enhanced CD62L population (*p* < 0.05) (Figures 4C,D). Secondary lymphoid organs like spleen and lymph nodes are known to serve as homing site for T_CM_ cell population (26, 27). Interestingly, we observed a drastic decrease (*p* < 0.05) in the T_CM_ cell (CD4^+^CD44^hi^CD62L^hi^) population obtained from spleen of ^Abx^BCG and ^Abx^L91 administered animals (Figures 4E,F). The results indicate that gut dysbiosis not only influence the ability of immune system at pulmonary sites, but also impairs the immunological memory at secondary lymphoid organs in vaccinated animals.

### Gut Dysbiosis Modulates Bacterial Composition and Thwarts Th1 Immune Response to Incapacitate Clearance of Mtb in Vaccinated Animals

**FIGURE 5:**
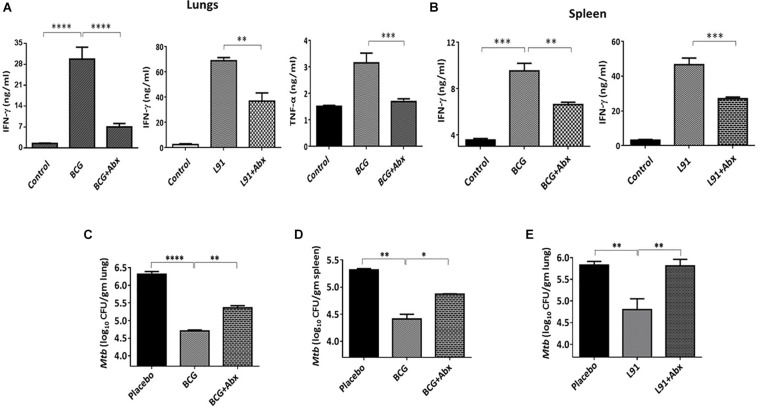
Gut dysbiosis impairs the efficacy of vaccines by inhibiting the differentiation of T cells. The cells isolated from the lungs and spleen of Abx treated and BCG vaccinated mice were cultured and *in- vitro* stimulated with *Mtb-*PPD for 48 h. Later, the culture supernatant was collected and T cell differentiation was monitored by estimating the yield of IFN-γ and TNF-α by ELISA in the cells isolated from **(A)** lungs and **(B)** spleen. The *Mtb* load in the **(C,E)** lungs and **(D)** spleen was enumerated by CFUs. Data shown as mean ± SEM are representative of two independent experiments (*n* = 3–4 animals/group). *p < 0.05, **p < 0.01, ***p < 0.001, and ****p < 0.0001.

Th1 cells are of utmost significance in the clearance of intracellular *Mtb*. Secretion of cytokines from these cells is an indicator of T cell activation. As a result, we monitored secretion of cytokines from the T cells of dysbiosis induced animals on stimulating with *Mtb-*specific PPD. Interestingly, there was a significant reduction in secretion of protective cytokines IFN-γ [lungs: BCG (p < 0.0001), L91 (*p* < 0.01); spleen: BCG (*p* < 0.01), L91 (*p* < 0.001)] and TNF-α [lungs: BCG (*p* < 0.001)] from the lungs and spleen of gut dysbiosis and vaccinated animals which indicated the suppressed immune response in these animals (Figures 5A,B). Our major aim was to monitor the effect of gut dysbiosis on intracellular survival of bacteria in vaccinated animals. We monitored the *Mtb* burden in lungs of dysbiosis induced animals and they showed a significantly higher (*p* < 0.01) *Mtb* burden in the lungs as compared to control animals (Figures 5C,E). Moreover, we also monitored the dissemination of *Mtb* to other organs in dysbiosis induced animals and interestingly, we observed a significant (*p* < 0.05) increment of bacterial burden in spleen isolated from dysbiosis induced animals (Figure 5D). Although the observed increment in bacterial burden in ^Abx^BCG and ^Abx^L91 groups was not too high, the results clearly suggested that disturbance of gut flora not only facilitates *Mtb* survival in vaccinated animals but also helps in the dissemination of *Mtb* to extra pulmonary organs like spleen thereby, further aggravating the disease.

Moreover, in order to have a better understanding of the changes in gut microbial composition after Abx treatment, we monitored the bacterial composition in the fecal sample by RT-PCR. Significant reduction of bifidobacterium [L91 (*p* < 0.0001); BCG (*p* < 0.0001)] and lactobacillus population [L91 (*p* < 0.001); BCG (*p* < 0.01)] and increased population of bacteroides [L91 (*p* < 0.0001); BCG (*p* < 0.0001)] was observed in ^Abx^BCG and ^Abx^L91 groups (Supplementary Figures 1A–C). To conclude that this suppressed immune system against *Mtb* was due to gut dysbiosis, fecal transplantation was done from healthy controls to ^Abx^BCG and ^Abx^L91 groups. Interestingly, there was a marked restoration of the proliferative ability of lungs T cells (Supplementary Figures 2A,B).

## Discussion

TB is a global health and economic burden, which has overtaken HIV/AIDS in causing maximum number of deaths worldwide. Importantly, this scenario occurs majorly in developing nations where TB burden is usually high. BCG is the only vaccine available against TB, which has proven to be inefficacious due to its inability to generate the long-lasting immunological memory in adults. Many reasons have been listed for the inefficacy of the BCG, such as malnutrition, excessive use of antibiotics, helminths infestation, and exposure to non-tuberculous mycobacteria. All these factors have recently gained importance due to their role in shifting the composition of gut microbiota, which in turn is responsible for modulating the immune response against various infectious diseases. Recently, we have shown that gut dysbiosis increases host susceptibility to TB, as indicated by increased *Mtb* burden in the lungs of mice and its dissemination to spleen and liver. In the current study, we have described the effect of gut dysbiosis on the efficacy of BCG and a lipidated peptide vaccine construct L91 which has shown promising results in imparting protection against TB.

Massive research on gut microbes has led to findings that reveal their correlation with various pathophysiological disorders. Use of antibiotics drives the change in composition of gut microbiota by dysregulation in physiological homeostasis that leads to severe disease conditions. Various immune cells such as macrophages, dendritic cells and T cells act as major players in deciding the disease outcome (28–30). Gut microbiota is known to play an imperative role in development and activation of T cell signaling pathways (31). Also, an imbalance in the number of T regulatory cells (Tregs) changes the frequency and composition of gut microbiota and *vice versa*. Gut microbiome plays a vital role in the development of CD4^+^ T cells, which act as major cells in protecting the host during *Mtb* infection (32). These reports suggest that gut microbiota has to be tuned and tamed in order to impart protection against invading pathogens. Our study describes an effect of gut dysbiosis on the efficacy of TB vaccines, which can be inferred from hampered activation of T cells and generation of memory T cells in vaccinated and *Mtb* infected animals.

For establishing association of gut microbiota with the efficacy of TB vaccines, gut dysbiosis model was induced by feeding the animals with a cocktail of Abx. The antibiotics regimen was selected as described previously (12). We selected antibiotics that were tested to be effective against broad spectrum flora. Furthermore, dose of antibiotics was carefully chosen that showed no effect on *Mtb* viability, even when administrated for 42 days (12). Fecal plating from these Abx-fed animals showed a change in composition and frequency of gut microbes and higher number of *Mtb* in the lungs. CD4^+^ T cells play a central role in imparting protection against *Mtb* (33, 34). CD4^+^ T cells secrete mainly IFN-γ and IL-2 that are required for CD8^+^ T cells development (35–37). CD8^+^ T cells depletion has shown to increase *Mtb* burden in lungs of mice (38). Consequently, keeping in mind the cardinal roles played by CD4^+^ T cells and CD8^+^ T cells during *Mtb* infection, the differentiation and activation of these cells was checked in BCG and L91 vaccinated animals infected with *Mtb*. Intriguingly, drastic decline in proliferation and activation markers was observed in vaccinated animals on gut dysbiosis.

A successful vaccine candidate elicits an enduring memory T cell response. In contrast, BCG is known to impart weak memory response and therefore fails to protect against the childhood but not adulthood manifestation of TB (39). Abx mediated gut dysbiosis in BCG vaccinated animals resulted in dampened central, effector, as well as resident memory T cell response, indicating that gut dysbiosis might be one of the factors responsible for weak immunological response in BCG vaccinated population. Recently, a third set of memory T cells has been identified that are named as resident memory T cells present mainly in non-lymphoid organs (40).

Antigen presentation takes place initially in the secondary lymphoid organs. It was found that Abx treatment suppressed the immunological response in these organs, as observed by hampered activation of T cells and decreased population of central memory T cells. IFN-γ producing Th1 cells play a vital role in autophagic pathways, which lead to clearance of bacteria (41). However, expression of protective cytokines IFN-γ and TNF-α was also found to be decreased in T cells isolated from Abx treated and vaccinated animals, suggesting that Abx mediated gut dysbiosis impairs the immune response against *Mtb*. Our results suggest that L91 is better inducer of IFN-γ on recall response. Previous published study has demonstrated that L91 vaccine shows better protective response than BCG in imparting protection against TB (17). This may be due to the limitation of mycobacteria to hamper the processing and presentation of antigens leading to subdued induction of Th1 response including IFN-γ secretion (42, 43). Immunodominant subunit vaccines such as L91 are attractive alternatives which can circumvent epitopes and can induce protective immunity against *Mtb*. Further, our data showed depletion of bifidobacterium and increased population of bacteroides. Bacteroides are known to enhance anti-inflammatory response (44). Furthermore, fecal transplants from healthy to gut disrupted animals restored the immunological response toward *Mtb*, confirming that gut flora plays an important role in modulating the TB vaccine efficacy. Across the globe, there occurs a variable gut microbiome in the population, which might correspond to variable efficacy of BCG in imparting protection against *Mtb*. This study implies that gut microbes such as bifidobacterium that are responsible for inducing inflammatory immune response during infection, can be harnessed for developing probiotics. Our lab is now working and collaborating further on the microbiome characterization and analysis through metagenomics for other upcoming studies. This study opens up new avenues in the field of adjunct therapy against *Mtb* by supplementing probiotics with BCG to bolster its potency against *Mtb*. This study also paves a way for augmenting the efficacy of vaccines by supplementing them with probiotics, which is an interesting line of investigation. Currently our laboratory is working on it.

## Data Availability Statement

All datasets generated for this study are included in the article/Supplementary Material.

## Ethics Statement

The animal study was reviewed and approved by the Institutional Animal Ethics Committee of IMTECH. Experiments were performed in accordance with the National Regulatory Guideline issued by Committee for the Purpose of Supervision of Experiments on Animals (No. 55/1999/CPCSEA), Ministry of Environment and Forest, Government of India.

## Author Contributions

JA conceived the idea and troubleshot the problems related to experiments. JA and SN designed the experiments, analyzed the data, and wrote the manuscript. SN, SM, DD, and NK performed the experiments.

## Conflict of Interest

The authors declare that the research was conducted in the absence of any commercial or financial relationships that could be construed as a potential conflict of interest.

## Abbreviations

Abx: antibiotics
BCG: Bacillus Calmette-Guérin
CFU: colony forming units
*Mtb*: *Mycobacterium tuberculosis;* TB tuberculosis
T_CM_: T central memory cells
T_EM_: T effector memory cells.
