# Supplementary material for: Gut Dysbiosis Thwarts the Efficacy of Vaccine Against Mycobacterium tuberculosis
Source: Front Immunol. 2020 May 19;11:726. doi: 10.3389/fimmu.2020.00726 (PMC7248201; doi:10.3389/fimmu.2020.00726)
Supplement: Supplementary file 1 [file Data_Sheet_1.pdf]

Supplementary figure 1

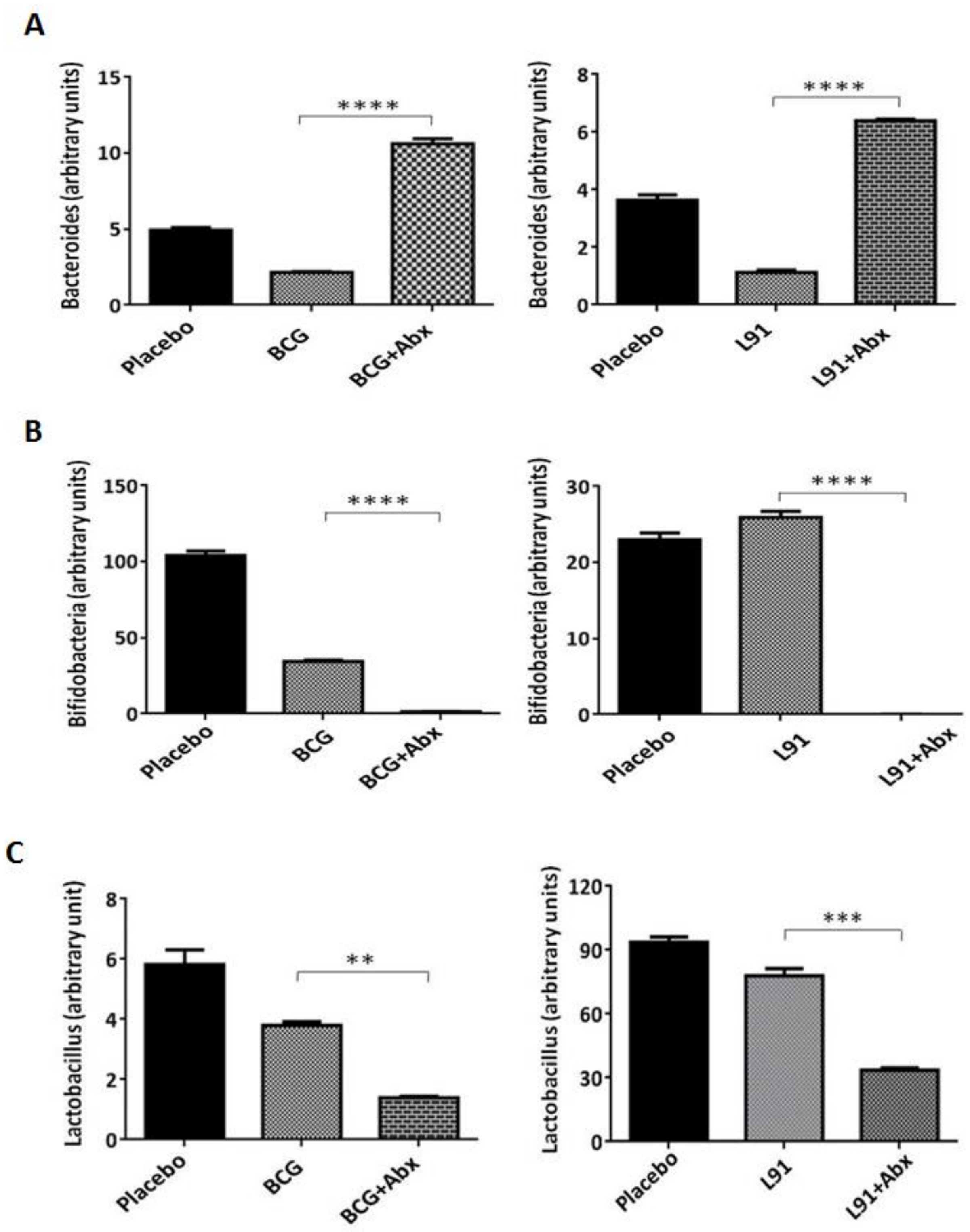

Supplementary figure 1. Modulation of the gut microbial architecture of the vaccinated animals on Abx treatment. The change in the gut microbial population by Abx treatment was monitored in the fecal samples of the BCG and L91 immunized animals by RT-qPCR. The genomic DNA was isolated and assessed for (A) bacteroides, (B) bifidobacterium (C) lactobacillus. Data shown as mean±SEM are representative of two independent experiments (n=3-4 animals/group). **p<0.01, ***p<0.001, ****p<0.0001.

Supplementary figure 2

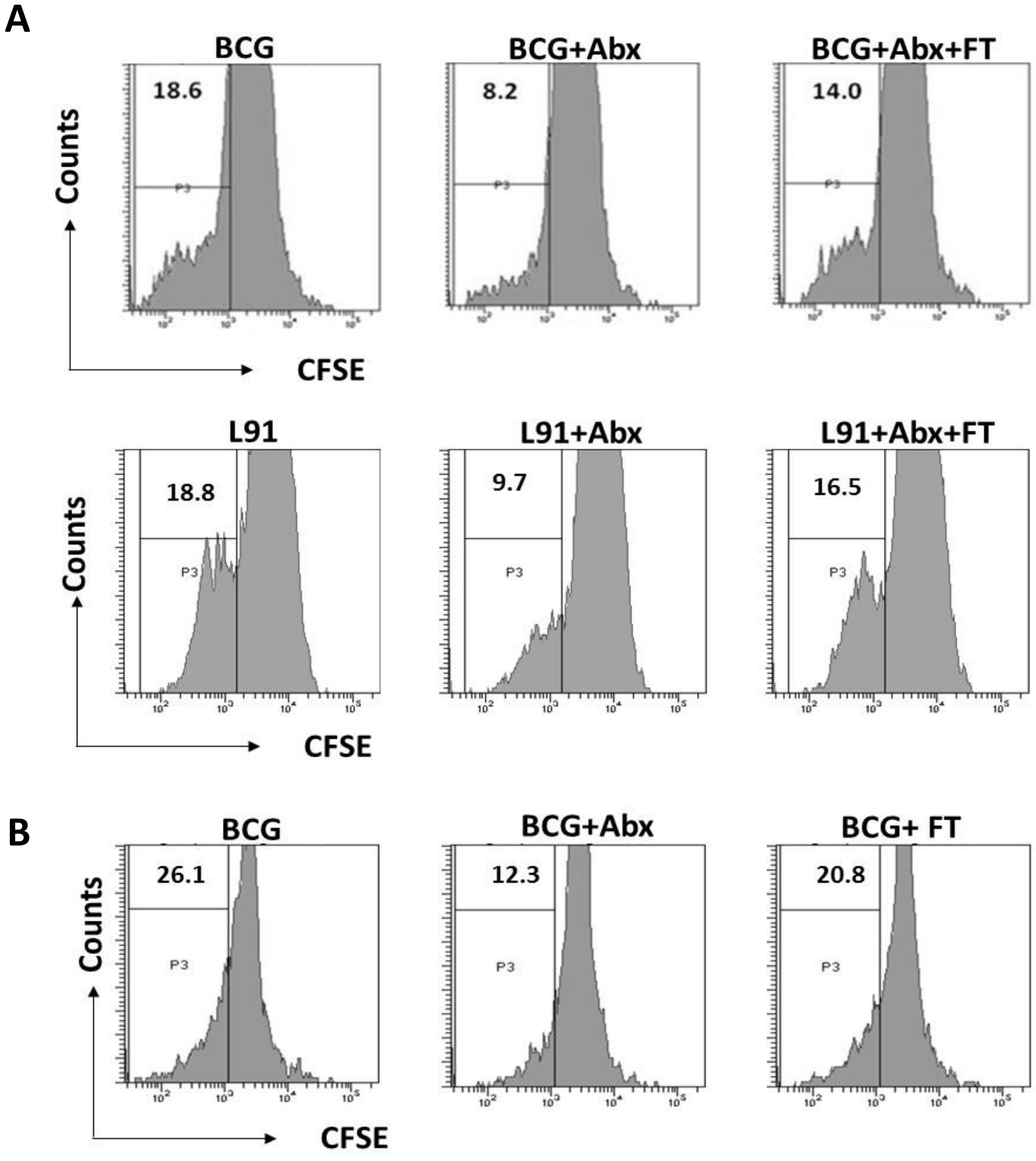

Supplementary figure 2. Fecal transplant restore the proliferation of T cells. The mice were vaccinated, as specified in Figure 2. The animals were fed five doses of fecal transplant, 15 days prior to their sacrifice. The cells isolated from the lungs of Abx treated and vaccinated mice were CFSE-labeled and cultured. The proliferation of (A) $CD4^+$ T cells; (B) $CD8^+$ T cells obtained from BCG and L91 vaccinated groups was monitored. Data represented as histograms are from two independent experiments (n=3-4 animals/group).

Supplementary figure 3

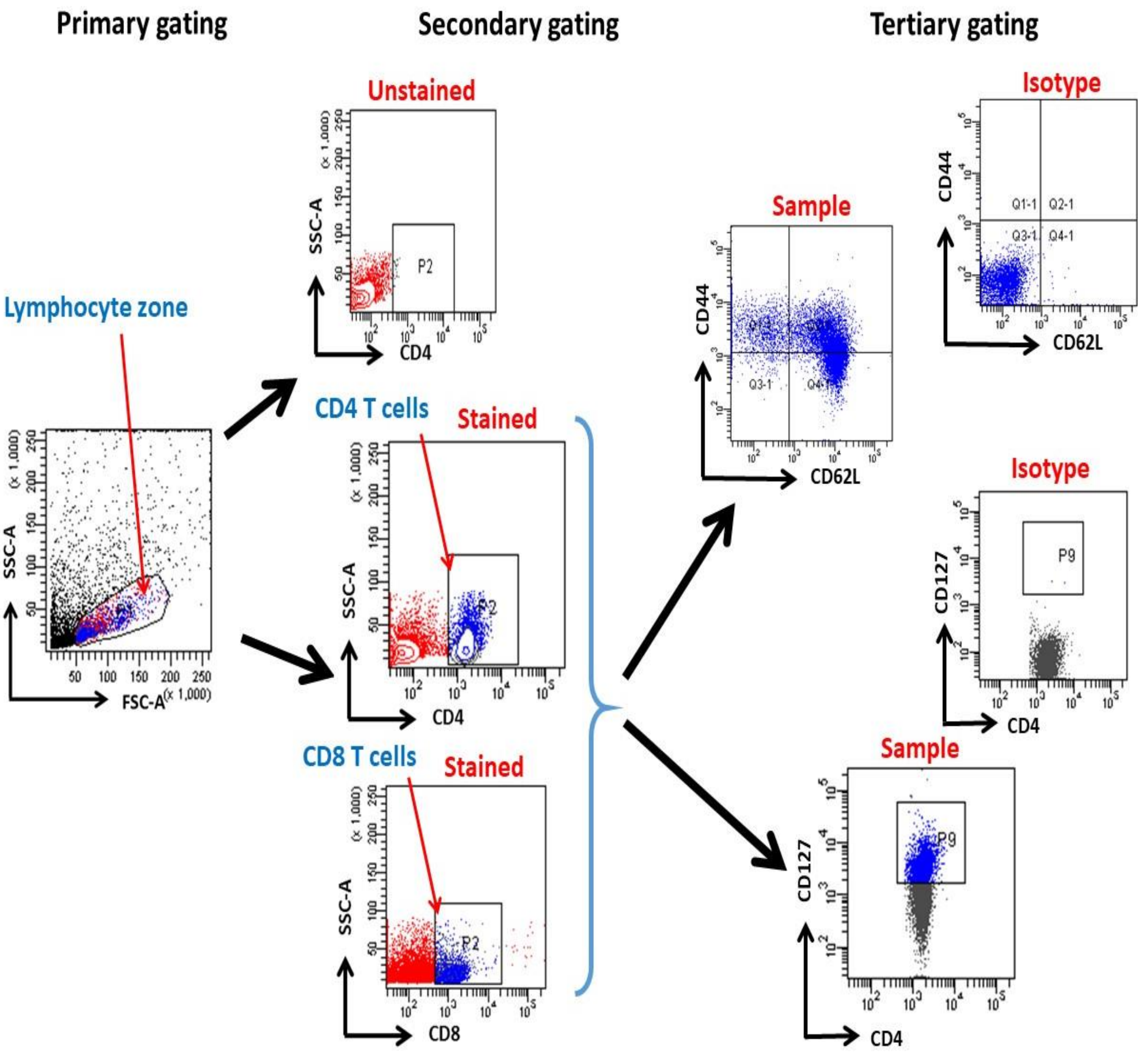

Supplementary figure 3. Gating strategy for monitoring the expression of central memory population ($CD44^{hi}CD62L^{hi}$) and effector memory population ($CD44^{hi}CD62L^{lo}$) of $CD4^{+}$ T cells and $CD8^{+}$ T cells. The primary gate was made on lymphocyte zone and secondary gate on SSC-A vs. either $CD4^{+}$ T cells ($CD4^{+}$/SSC-A) or $CD8^{+}$ T cells ($CD8^{+}$/SSC-A) population. The tertiary gate shows the expression of CD44, CD62L (CD44/CD62L; double gating) and CD127 ($CD127^{+}$/$CD4^{+}$). The isotype-matched control shows only double negative $CD44^{-}CD62L^{-}$ population (CD44/CD62L; double gating) and no CD127 positive population ($CD127^{+}$/$CD4^{+}$).

Supplementary figure 4

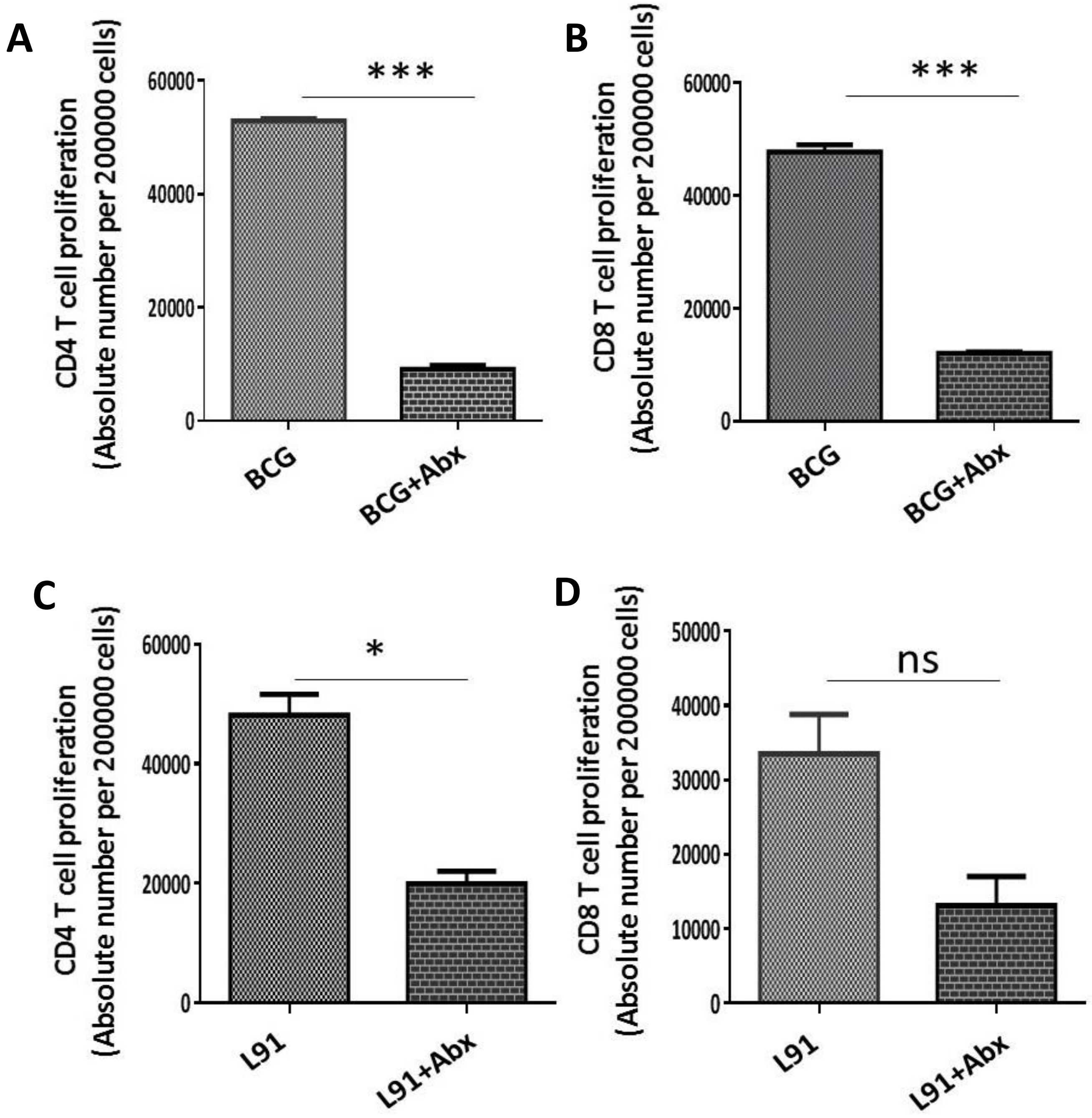

Supplementary figure 4. Gut dysbiosis in vaccinated animals hampered the proliferation of T cells. After 21 days of *Mtb* exposure, the lungs were isolated from the BCG and L91 vaccinated and Abx treated mice. Single cell suspensions of lung cells were prepared and equal number of cells in both groups were stained with CFSE/efluor dye. Subsequently, the cells were *in vitro* stimulated with PPD for 48h. Later, the cells obtained from (A-B) BCG and (C-D) L91 vaccinated mice were gated on (A, C) $CD4^{+}$ T and (B, D) $CD8^{+}$ T cells and monitored for the proliferation by CFSE/efluor dye dilution assay. Bar graphs represents absolute number of proliferating cells per 200000 cells analysed. Data shown as mean±SEM are the representative of 2 independent experiments (n=3-4 animals/group). *p<0.05, ***p<0.001.

Supplementary figure 5

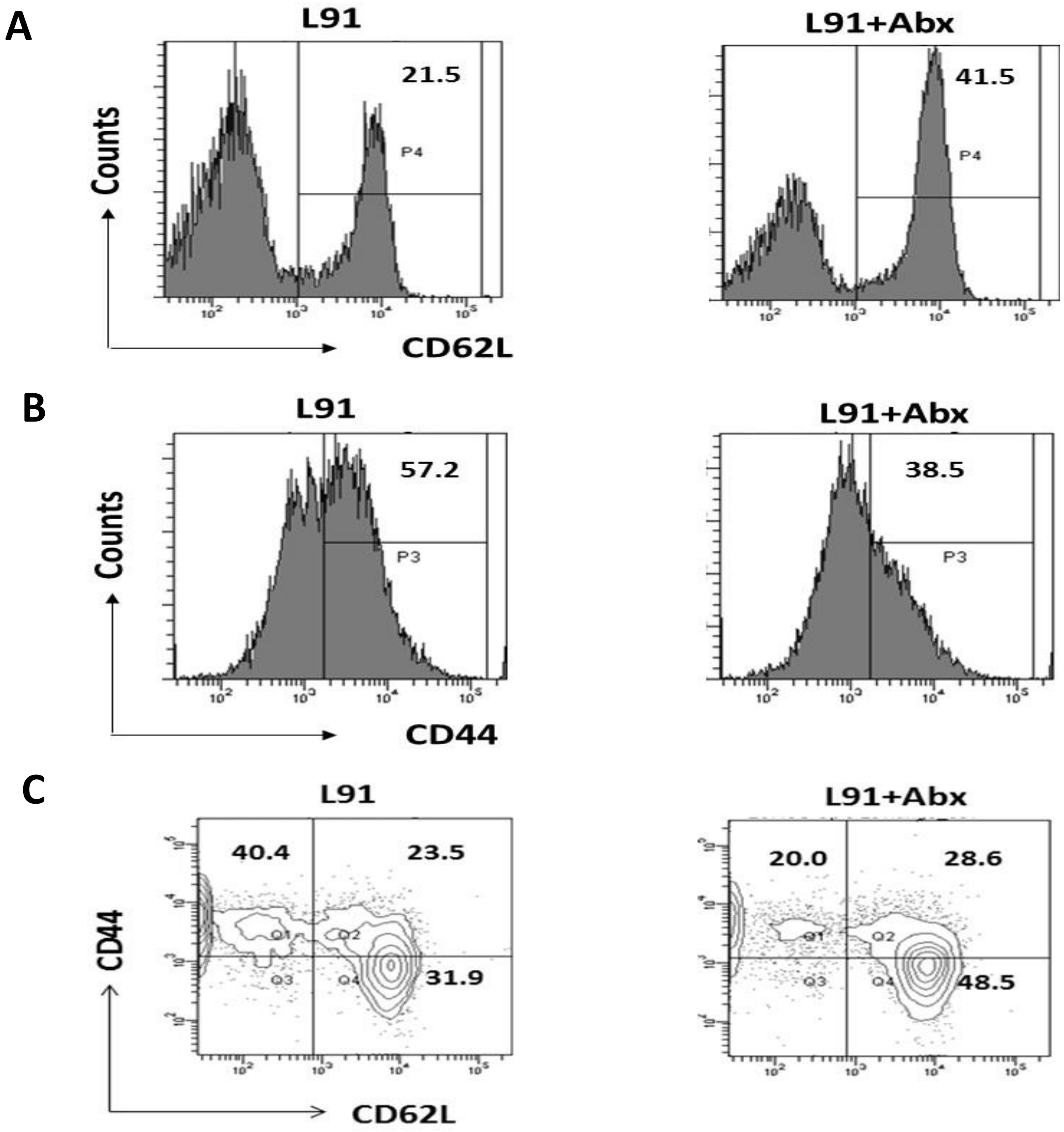

Supplementary figure 5. Abx mediated gut dysbiosis impaired the activation and generation of memory T cells in lungs. The cells isolated from the lungs of L91 vaccinated and Abx treated mice were cultured and *in- vitro* stimulated with L91 (1 nmol) for 48h. After 48h, the cells were harvested, stained and gated on $CD4^{+}$ T cells and assessed for the (A-B) activation markers CD44 and CD62L and (C) effector memory marker $CD44^{hi}CD62L^{lo}$ (T_{EM}). Values in the inset signify percent population. Data shown is representative of one independent experiment (n=3-4 animals per group).
